# Persistence of Naturally Acquired and Functional SARS-CoV-2 Antibodies in Blood Donors One Year after Infection

**DOI:** 10.3390/v14030637

**Published:** 2022-03-18

**Authors:** Verena Nunhofer, Lisa Weidner, Alexandra Domnica Hoeggerl, Georg Zimmermann, Natalie Badstuber, Christoph Grabmer, Christof Jungbauer, Nadja Lindlbauer, Nina Held, Monica Pascariuc, Tuulia Ortner, Eva Rohde, Sandra Laner-Plamberger

**Affiliations:** 1 Department for Transfusion Medicine, University Hospital of Salzburg (SALK), Paracelsus Medical University (PMU) Salzburg, Müllner-Hauptstraße 48, 5020 Salzburg, Austria; v.nunhofer@salk.at (V.N.); a.hoeggerl@salk.at (A.D.H.); c.grabmer@salk.at (C.G.); n.lindlbauer@salk.at (N.L.); n.held@salk.at (N.H.); m.pascariuc@salk.at (M.P.); e.rohde@salk.at (E.R.); 2 Austrian Red Cross, Blood Service for Vienna, Lower Austria and Burgenland, Wiedner Hauptstraße 32, 1040 Vienna, Austria; lisa.weidner@roteskreuz.at (L.W.); christof.jungbauer@roteskreuz.at (C.J.); 3 Team Biostatistics and Big Medical Data, IDA Lab Salzburg, PMU Salzburg, Strubergasse 16, 5020 Salzburg, Austria; georg.zimmermann@pmu.ac.at; 4 Research and Innovation Management, PMU Salzburg, Strubergasse 16, 5020 Salzburg, Austria; 5 Department of Psychological Assessment, Institute of Psychology, Paris-Lodron-University of Salzburg, 5020 Salzburg, Austria; natalie.badstuber@plus.ac.at (N.B.); tuullia.ortner@plus.ac.at (T.O.); 6 Spinal Cord Injury and Tissue Regeneration Centre Salzburg, PMU Salzburg, Strubergasse 21, 5020 Salzburg, Austria

**Keywords:** SARS-CoV-2, COVID-19, long-COVID, seroprevalence, antibodies, blood donation, AB0 blood groups

## Abstract

The developmental course of antibodies produced after a SARS-CoV-2 infection has been insufficiently investigated so far. Therefore, the aim of this study was to investigate the dynamics of SARS-CoV-2 antibody levels against the viral nucleocapsid- and spike-protein among Austrian blood donors as a representative group of a supposedly healthy population within the first year after a SARS-CoV-2 infection. The impact of age, sex, vaccination status, AB0-blood group and awareness about the infection was evaluated. Our study shows that the level of anti-N antibodies is declining, while anti-S antibody levels remain stable. Antibodies detected were functional in vitro. Age, sex and blood group do not influence antibody dynamics. However, blood group AB shows significantly lower antibody levels and in vitro functionality compared to other blood groups. Our data reveal that one out of five individuals was not aware of a previous SARS-CoV-2 infection and that the disease course neither affects the level of antibody production nor the in vitro functionality. We also found that 14% of participants show persisting COVID-19-related symptoms for up to nine months. Our results provide valuable insights into the dynamics of the immune response after a SARS-CoV-2 infection in a representative cohort of adult blood donors in Central Europe.

## 1. Introduction

Since December 2019, severe acute respiratory syndrome coronavirus 2 (SARS-CoV-2) has been a major health issue worldwide. Due to its fast spreading mode, the World Health Organization (WHO) declared a pandemic state in March 2020 [1]. Despite the availability of vaccination since the beginning of 2021, several variants of SARS-CoV-2 have recently led to further pandemic waves [2,3]: In spring 2021, the SARS-CoV-2 variant B1.617.2 spread globally, causing further damage to the already seriously struck health care systems. Currently governments are trying to prevent further spreading of the even more infectious variant B.1.1.529 [4].

SARS-CoV-2 is an enveloped single stranded RNA-virus of zoonotic origin belonging to the family of beta-coronaviruses. It is the causative agent of coronavirus disease 2019 (COVID-19), which resembles severe acute respiratory syndrome (SARS) and Middle East respiratory syndrome (MERS) [5,6]. Even though the majority of SARS-CoV-2 positively tested individuals are either asymptomatic or develop rather mild, flu-like symptoms, an infection might turn into a serious health problem [7,8]. Furthermore, many individuals suffer from prolonged symptoms (>35 weeks to recovery [9]) after a SARS-CoV-2 infection, termed post-acute sequelae of SARS-CoV-2 (PASC) or “long-COVID” [10].

Even though various vaccines against SARS-CoV-2 were proven to be safe and efficient [11,12], frequent cases of vaccine breakthroughs have been reported. [13]. Furthermore, there is an increasing number of reports concerning the reinfection of people previously infected with SARS-CoV-2 [14,15,16]. The actual numbers of SARS-CoV-2 infections are difficult to estimate due to asymptomatic or mild disease courses. This is a challenge for the prevention of disease dissemination as infected but pauci-/asymptomatic individuals further spread the virus without noticing. Another issue is the longevity of antibodies directed against SARS-CoV-2. As reviewed in Murchu et al. [17], specific IgG antibodies against SARS coronavirus, which was endemic in China in 2003, were found up to two years after an infection. Similar data are available for infections with MERS coronavirus [8,17]. To date, studies indicate that specific IgG antibodies against SARS-CoV-2 spike-protein are detectable up to 12 months after an infection [18,19]. A further study estimates antibody levels associated with protection against reinfection are likely to last up to two years, and antibody levels protecting against severe reinfection are present for several years [20]. Obtaining more data on infection rates and antibody dynamics, taking into account factors that may influence antibody production, could help to determine more accurate seroprevalence rates and to examine the developmental course of antibodies produced in more detail. This could support national authorities and health care systems to make appropriate decisions to fight the virus dissemination.

Thus, the aims of this study were (i) to investigate the developmental course of SARS-CoV-2 antibodies directed against the viral nucleocapsid-proteins (N-protein), which are produced after a SARS-CoV-2 infection only, and (ii) to determine the dynamics and in vitro functionality of antibodies directed against the viral spike-proteins (S-protein), which are produced after infection and vaccination. Our study focused on the first year after a SARS-CoV-2 infection and examined the influence of different factors (age, sex, vaccination status, AB0 blood group and awareness regarding the infection) on antibody development in blood donors as a representative group of the supposedly healthy adult Austrian population.

## 2. Materials and Methods

### 2.1. Ethical Statement

In this study, human residual serum for routine laboratory diagnostics as conducted in the course of standard blood donation work-up, according to European and local regulations, was used. All donors of the samples gave signed informed consent on the use of leftover material for research purposes. For detailed analysis of the developmental course of SARS-CoV-2 antibodies, blood donors who tested serologically positive for anti-N total antibodies were invited to take part in this study. The ethical committee of the Federal State of Salzburg, Austria appraised and waived the study conducted (ethical votum number 1004/2021). The work described has been carried out in accordance with the 1964 Helsinki Declaration and its later amendments or comparable ethical standards. Samples were processed anonymously to protect privacy of each donor.

### 2.2. Sample Collection and Study Design

For the determination of the seroprevalence rate of anti-SARS-CoV-2 antibodies, we studied the serum samples of 51,797 blood donations, which were collected in the course of voluntary, non-remunerated whole blood donations in the Federal State of Salzburg, Austria from 5 June 2020 until 31 December 2021. As already described in our previous study [21], all donors had a brief health screening and completed a written questionnaire, including an informed consent on pathogen screening as a standard part of the blood donation process. No further preselection of sample material was conducted. Concerning demographics, it is important to note that children (<18 years) and individuals older than 70 years were not included as individuals of these age groups are not admitted to regular blood donation. 

After a positive screening result for SARS-CoV-2 anti-N total antibodies, providing negative screening for other infectious disease parameters tested (serological and molecular biological screening for HIV, HBV, HCV, HAV, PB19, Syphilis and WNV as a part of the standard work-up of each blood donation), the donors were contacted by our study team. Recruitment of participants started in December 2020. In total, 120 seropositive blood donors willing to participate could be included in this study after signing informed consent. Blood samples (2 × 5 mL) were collected 3, 6 and 9 months after the seropositive initial blood donation, equaling the time span of the first year after a SARS-CoV-2 infection.

### 2.3. Questionnaires

All individuals included in this study were invited to participate in two online surveys containing questions concerning the course of the SARS-CoV-2 infection, the vaccination status, persisting symptoms related to the SARS-CoV-2 infection and other health-associated issues such as general health status and known comorbidities. These online questionnaires were to be filled in 3 and 9 months after the initial blood donation. The questionnaires were administered using Lime Survey (https://www.limesurvey.org, accessed on 2 March 2022).

### 2.4. Serological Testing

Four different serological screening approaches were applied: The Elecsys Anti-SARS-CoV-2 (ACOV2) total antibody electrochemiluminescence immunoassay (ECLIA, Roche Diagnostics, Basel, Switzerland) was applied to screen for SARS-CoV-2 anti-N total antibody (including IgM, IgG and IgA) using a cobas8000-e801 device (Roche Diagnostics) according to manufacturer’s instructions. In this semi-quantitative test, a recombinant protein of the viral nucleocapsid (N) antigen is used to determine antibodies against SARS-CoV-2. The sensitivity and specificity given by the manufacturer is 100% and 99.81%, respectively. The results of this screening approach are based on the sample signal to cut-off ratio, with values <1.0 corresponding to negative results and values ≥1.0 corresponding to positive results. According to the manufacturer, this screening assay is able to detect but not discriminate all SARS-CoV-2 variants known so far, including also the variants delta and omicron.

The ECLIA Elecsys Anti-SARS-CoV-2 S (Roche Diagnostics) was conducted on the cobas8000-e801 and uses the receptor binding domain (RBD) of the spike-protein (S-protein) of SARS-CoV-2 as target. The sensitivity given by the manufacturer is 98.8%, the specificity is 99.9%. Results are quantitative and expressed as IU/mL. The cut-off for samples considered positive is ≥0.80 IU/mL. As stated by the manufacturer 1 IU/mL is equivalent to 0.972 BAU/mL. The Wantai SARS-CoV-2 Ab ELISA (Wantai Biological Pharmacy, Beijing, China), which uses the RBD as a target too, was performed on the BEP III (Siemens Health Diagnostics GmbH, Eschborn, Germany) or ETI-Max 3000 (DiaSorin S.p.A., Saluggia, Italy) platform. As published earlier, the Wantai SARS-CoV-2 Ab ELISA shows a sensitivity of 98% [22]. The results are expressed as ratios of the cut-off. Ratios greater than 1 are considered positive.

In order to detect neutralizing antibodies against SARS-CoV-2 that interrupt the interaction between the receptor binding domain (RBD) of the viral spike glycoprotein with the human angiotensin converted enzyme 2 (ACE-2) cell surface receptor, the SARS-CoV-2 surrogate virus neutralization test (sVNT) (GenScript, Piscataway Township, NJ, USA) was used. In brief, this assay tests whether antibodies against SARS-CoV-2 present in the human serum tested are able to block the protein–protein interaction between a horseradish peroxidase conjugated recombinant viral RBD protein and the human ACE-2, which is coated on 96-well plates. Results are expressed as percent signal inhibition (=net OD450 sample value/OD value of negative control × 100). According to the manufacturer, this assay shows 100% sensitivity and 100% specificity when compared to plaque reduction neutralization assay. Signal inhibition ≥30% is considered as positive, indicating that neutralizing antibodies against SARS-CoV-2 are detected. All values <30% are considered as negative, indicating that not sufficient neutralizing antibodies could be detected.

### 2.5. Data Collection and Statistical Analysis

In a primary analysis, the data were summarized descriptively, using counts and percentages, or medians and interquartile ranges, depending on the scale of the variable. The initial analysis was performed using the full dataset including all participants with two complete questionnaire datasets and laboratory data of the 3 pre-specified points in time (*n* = 120). In a sensitivity analysis, several subsets of the data stratified according to the time between the known SARS-CoV-2 infection and initial blood donation were considered. For assessing statistical significance of between-group differences (e.g., between different blood groups) and for comparing the antibody dynamics over time between groups (i.e., testing for group-time interactions), nonparametric ANOVA-type tests were used. These tests are implemented in the nparLD package [23] in the statistical software environment R version 3.5.1 [24]. For comparisons between two groups (e.g., vaccinated yes/no) at one particular point in time, Wilcoxon-Mann-Whitney test was applied. The reason for using nonparametric tests is that they are based on ranks and, hence, are more robust in the case of highly asymmetric distributions and outliers compared to the classical parametric approaches. Since the list of grouping factors for the between-group comparisons has been specified in advance, no adjustment for multiplicity was applied. For all statistical tests, the two-sided significance level was set to 5 percent. All analyses were conducted using the statistical software R v3.5.1. [24]. 

## 3. Results

### 3.1. Infection-Acquired Natural SARS-CoV-2 Antibodies Are Detectable and Functional In Vitro for at Least One Year

**Figure 1:**
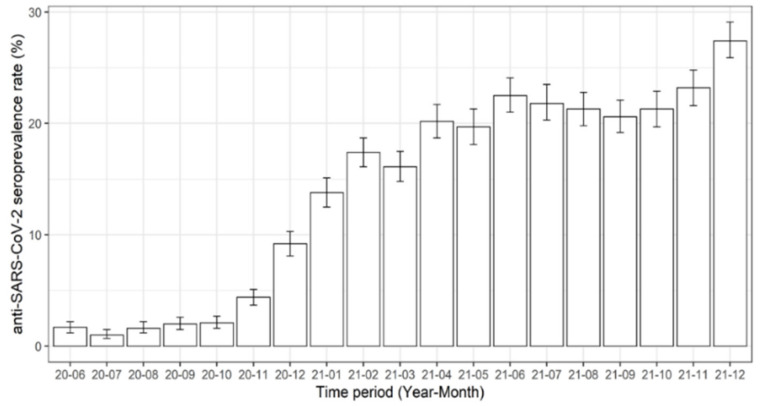
Seroprevalence rates of blood donations screened positive for SARS-CoV-2 anti-N total antibodies in Salzburg, Austria. Time is indicated as year-month.

There were 51,797 regular blood donations screened for SARS-CoV-2 anti-N total antibody levels between June 2020 and December 2021. This type of antibody is produced after a SARS-CoV-2 infection but not after a vaccination, which allows discrimination between vaccinated and unvaccinated individuals. While in June 2020 the seroprevalence rate was 1.7%, this rate was constantly rising over time and reached 27.4% at the end of December 2021 (Figure 1). 

Blood donors who screened positive for SARS-CoV-2 anti-N total antibodies were invited to participate in the present study. To date, 120 participants have been included, with only six individuals reporting known pre-existing conditions (allergic asthma, hypercholesterinaemia, depression, hypothyroidism and hypertension). Therefore, the included participants represent a supposedly healthy subgroup of the Austrian adult population. 

**Figure 2:**
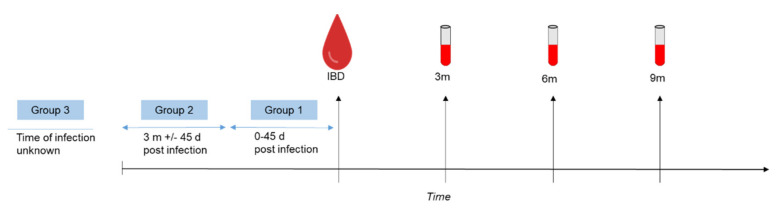
Study workflow and grouping of study participants. Study participants were asked for blood samples 3, 6 and 9 months (3 m, 6 m, 9 m) after the initial blood donation (IBD) that was screened positive for SARS-CoV-2 anti-N total antibodies.

**Figure 3:**
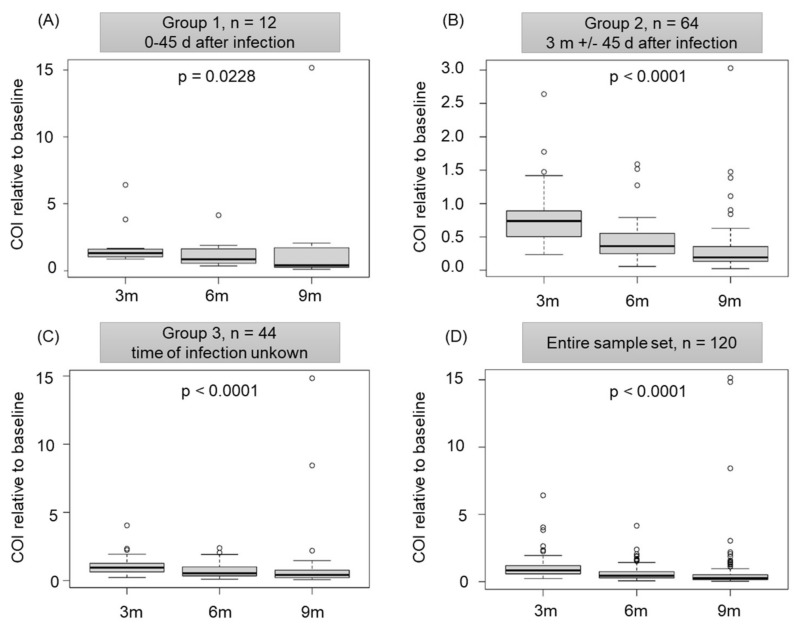
SARS-CoV-2 anti-N total antibody levels significantly decline but are detectable one year after an infection. (**A**–**C**) The participants were divided into three groups according to the time of infection and time of blood donation (group 1: 0–45 days post SARS-CoV-2 infection, group 2: 3 months (3 m) +/− 45 days post SARS-CoV-2 infection, group 3: no information about the time of infection with SARS-CoV-2 available). (**D**) Decline in anti-N total antibody levels for all 120 study participants. Data shown are cut-off indices (COI) of SARS-CoV-2 anti-N total antibodies relative to the baseline signal (=COI value of the initial donation) 3, 6 and 9 months (m) after the initial blood donation. *p* = *p*-value for time trend.

Additional blood samples were taken 3, 6 and 9 months after the initial blood donation and SARS-CoV-2 anti-N total antibody levels were measured. For statistical analysis, the participants were divided into three subgroups (Figure 2): Group 1 (*n* = 12) donated blood up to 45 days after an infection, while group 2 (*n* = 64) donated blood 3 months +/− 45 days after an infection with SARS-CoV-2. Participants belonging to group 3 (*n* = 44) either did not know about their infection or were not able to give information about the time of infection. All groups showed significantly declining levels of SARS-CoV-2 anti-N total antibodies (*p* = 0.0288 for group 1 and *p* < 0.0001 for groups 2 and 3, Figure 3A–C). Despite this decline, almost all study participants showed detectable levels of anti-N antibodies. Three months after the initial blood donation only one person was screened as negative, while after 6 months, this was four individuals, and after 9 months, six individuals were screened negative for SARS-CoV-2 anti-N total antibodies. Due to these similar trends within subgroups, and since the analysis of the entire sample set (*n* = 120) also revealed a significant decline in total anti-N antibodies (Figure 3D), the entire sample was considered for further analysis.

As a next step, we grouped the study participants according to their vaccination status into two subgroups: unvaccinated (*n* = 46) and vaccinated (*n* = 74, at least one vaccination against SARS-CoV-2, irrespective of the vaccine used). In both groups, the levels of SARS-CoV-2 anti-N total antibodies were comparable for each point in time investigated. These antibody levels substantially declined, while the levels of antibodies against the viral S-protein remained rather stable over time, as demonstrated by IgG ELISA and quantitative ECLIA (Table 1). As expected, quantitative amounts of SARS-CoV-2 antibodies directed against the viral S-protein are substantially lower for unvaccinated (35.6 IU/mL 3 months, 42.1 IU/mL 6 months and 69 IU/mL 9 months after the initial blood donation) than for vaccinated participants (107 IU/mL for 3 months after the initial blood donation, ≥2500 IU/mL for later points in time). The in vitro functionality of the antibodies detected was investigated using a surrogate virus neutralization test. As shown in Table 1, unvaccinated individuals showed a stable median inhibition rate of 61.9% (3 months after the initial blood donation), 59.8% (6 months after the initial blood donation) and 63.4% (9 months after the initial blood donation). Even though there was no significant decline for in vitro functionality over time within the group of unvaccinated, it is important to note that already 3 months after the initial blood donation, three individuals of that group showed negative and a further eight individuals borderline results for in vitro functionality. Nine months after the initial blood donation, these numbers increased to 4 negative and 12 borderline results, indicating a trend towards declining in vitro functionality within the first year after a SARS-CoV-2 infection. Vaccinated individuals showed substantially higher in vitro inhibition rates (79.2%, 95.6% and 94.7% over time). None of the vaccinated individuals showed negative or borderline results for in vitro functionality for the points in time investigated.

### 3.2. The Decline in Anti-N Antibodies Is Independent of Sex and Age

**Figure 4:**
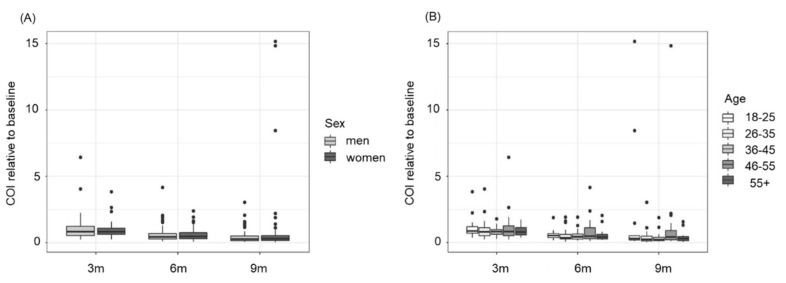
Decline in SARS-CoV-2 anti-N total antibody levels is not significantly dependent on sex or age. (**A**) No significant difference could be observed between women (*n* = 56) and men (*n* = 64) regarding antibody dynamics over time (3, 6 and 9 months (m) post initial blood donation). (**B**) Analysis according to different age groups (age 18–25: *n* = 15; age 26–35: *n* = 27; age 36–45: *n* = 23; age 46–55: *n* = 33; age 56+: *n* = 22) revealed no significant differences over time. Data shown are cut-off indices (COI) of SARS-CoV-2 anti-N total antibodies relative to the baseline signal (=COI value of the initial blood donation).

We analysed the influence of sex and age on the development of total anti-SARS-CoV-2 antibody levels directed against the viral N-protein. Our data did not reveal any significant dependence on sex (*p*-value between women and men over time = 0.5013, Figure 4A) or age (*p*-value over time = 0.2768, Figure 4B).

### 3.3. Blood Group AB Shows Significantly Lower Levels and In Vitro Functionality of SARS-CoV-2 Antibodies

**Figure 5:**
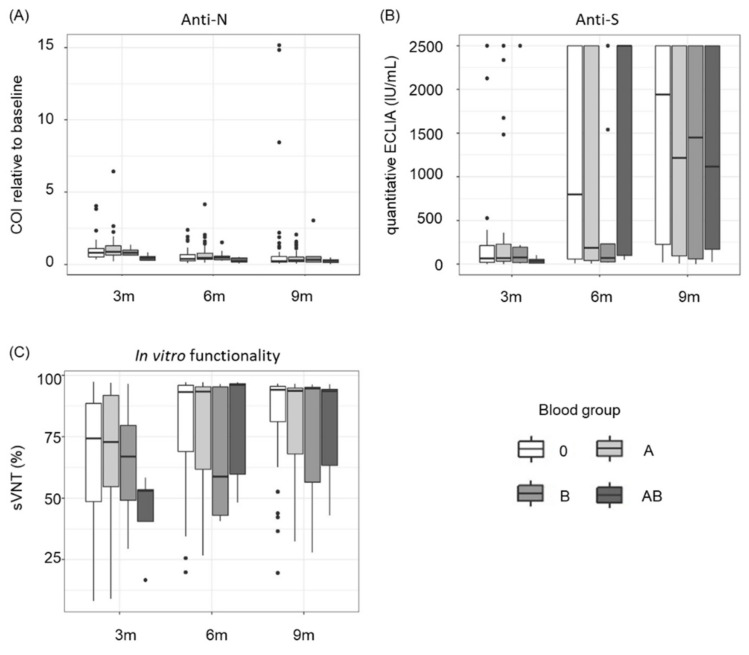
Influence of AB0 blood groups on SARS-CoV-2 antibody dynamics 3, 6 and 9 months after the initial blood donation. (**A**) Blood group AB shows significantly lower cut-off indices (COI) for anti-N overall compared to other blood groups (*p* = 0.0335). Data shown are COI of SARS-CoV-2 anti-N total antibodies relative to baseline signal. (**B**) Blood group AB shows substantially lower levels of anti-S antibodies 3 months post initial blood donation and (**C**) lower in vitro functionality of antibodies three months post initial blood donation. Data shown are antibody concentrations in IU/mL for (**B**) and inhibition rates given in percent for (**C**).

The distribution of AB0 blood groups among the participants of the present study reflects the normal AB0 blood group distribution in Austria (according to the Austrian Red Cross, https://www.roteskreuz.at/blutspenden/wissenswertes-zum-blut?gclid=EAIaIQobChMIj_2wz4rm9QIVwwyLCh0-4wjZEAAYASAAEgI0s_D_BwE, accessed on 15 February 2021) and is as follows: blood group A (*n* = 54, 45%), blood group B (*n* = 9, 7.5%), blood group 0 (*n* = 50, 41.7%) and blood group AB (*n* = 7, 5.8%). Overall, blood group AB donors showed significantly lower levels of anti-N antibodies compared to other blood groups (*p* = 0.0335) (Figure 5A). We also observed substantially lower concentrations of antibodies directed against the viral S-protein in blood group AB 3 months after the initial blood donation (Figure 5B). This effect disappeared over time, which can be explained by rising vaccination rates (3 months after the initial blood donation: 2/7 AB blood donors were vaccinated, 9 months post initial blood donation: 5/7 were vaccinated). Our data further reveal that the in vitro functionality of the antibodies is substantially lower 3 months after the initial blood donation for blood group AB (Figure 5C). Again, this effect disappeared for later points in time, most likely due to vaccination.

### 3.4. Asymptomatic and Symptomatic COVID-19 Disease Course Leads to a Similar Antibody Response

**Figure 6:**
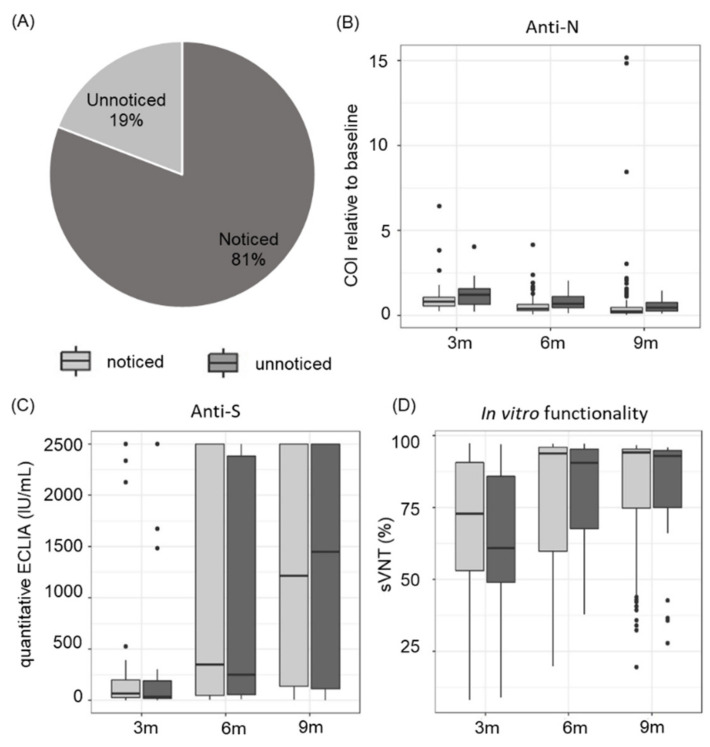
Asymptomatic and symptomatic COVID-19 disease course induces comparable antibody levels and in vitro functionality. (**A**) In total, 19% of SARS-CoV-2 infections were unnoticed. (**B**) Total anti-N antibody levels show comparable levels and decline over time for both types of disease course. (**C**) Quantitative analysis reveal similar concentrations for anti-S antibodies. (**D**) The in vitro functionality of anti-SARS-CoV-2 antibodies over time is comparable after asymptomatic and symptomatic disease course. Data shown are cut-off indices (COI) of SARS-CoV-2 anti-N total antibodies relative to baseline signal for (**A**), anti-S antibody concentration in IU/mL for (**B**) and in vitro inhibition rates given in percent for (**C**).

We further examined whether study participants were aware of their SARS-CoV-2 infection and found that 19% of all infections were unnoticed, indicating either an asymptomatic or a rather mild disease course with symptoms not being assigned to a SARS-CoV-2 infection (Figure 6A). Comparing anti-N total antibody dynamics of noticed and unnoticed disease course, no significant difference was observed (Figure 6B). The anti-S antibody titres were also comparable (Figure 6C). Furthermore, the in vitro functionality of antibodies directed against SARS-CoV-2 was similar in both groups (Figure 6D).

### 3.5. COVID-Related Symptoms Last up to 9 Months in Healthy Blood Donors

**Figure 7:**
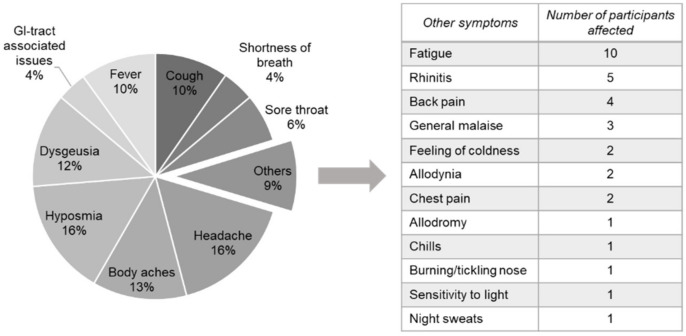
Symptoms experienced by blood donors during a symptomatic SARS-CoV-2 infection. Most commonly reported symptoms are shown on the left, other symptoms experienced are listed on the right.

A symptomatic SARS-CoV-2 infection was reported by 81% of our study participants. None of them reported hospitalization. Symptoms reported were headache (16%), hyposmia (16%), body aches (13%) and dysgeusia (12%), followed by cough and fever (both 10%). A total of 6% experienced a sore throat, 4% suffered from shortness of breath and issues concerning the GI-tract, and 9% of the study participants stated other symptoms such as fatigue, rhinitis and back pain (Figure 7).

**Figure 8:**
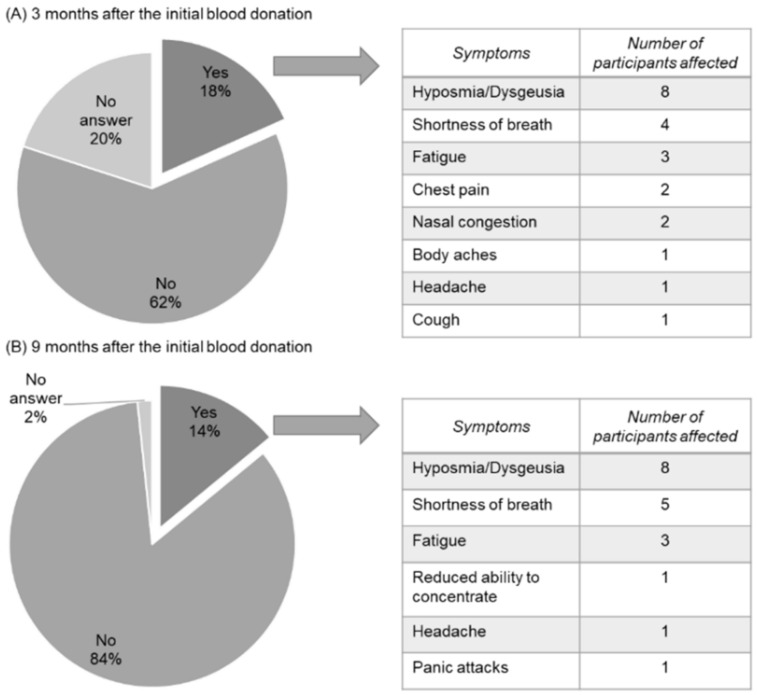
Long-term COVID-19-related symptoms among blood donors. (**A**) Three months after blood donation, 18% of the study participants reported suffering from symptoms related to a previous SARS-CoV-2 infection. This number decreased to 14% nine months after the initial blood donation (**B**).

While Taquet et al. [10] reported 30% long-COVID symptoms among COVID-19 patients, 18% of our study participants who noticed the infection still showed symptoms 3 months post initial blood donation: Eight individuals reported hyposmia or dysgeusia, four noticed a shortness of breath and a further three suffered from fatigue. Other symptoms reported were chest pain and nasal congestion (two individuals) and headache, body aches and cough (one person each). Nine months post initial blood donation, equalling about one year after the infection with SARS-CoV-2, 14% of the study participants still reported symptoms due to COVID-19, with the main health issues being hyposmia and dysgeusia (eight individuals), shortness of breath (five participants) and fatigue (three participants) (Figure 8). We also asked asymptomatic participants, who did not notice their infection, whether they had had, or had still, symptoms they would assign to a SARS-CoV-2 infection. None of them reported any symptoms for any point in time.

## 4. Discussion

The screening for SARS-CoV-2 anti-N total antibodies of 51,797 Austrian blood donations revealed a constantly rising seroprevalence rate, starting from 1.7% in June 2020 and peaking at 27.4% in December 2021. These data reflect the actual infection numbers with SARS-CoV-2 among supposedly healthy individuals at the age of 18–70 years in Salzburg, Austria, as screening for infection-acquired antibodies was conducted. This method further allowed the screening for all variants of SARS-CoV-2 known so far but not their discrimination as, to date, mutations mainly affect the viral S-protein. The data obtained in this study reflect the infection-induced seroprevalence rates found for blood donors in many European countries as reviewed by Vaselli et al., [25], but also other countries with comparable health care systems such as the USA [26] and the UAE [27]. Blood donors positively screened for naturally acquired SARS-CoV-2 anti-N total antibodies were invited to participate in the present study. Even though we observed declining levels of anti-N antibodies within the first year after an infection, these antibodies were still detectable in 95% of our study participants 9 months after the initial blood donation (114 of 120 participants). A similar decline in anti-N antibodies within the first year after an infection was also observed in other studies investigating healthcare workers and COVID-19 patients [28,29,30].

Vaccinated as well as unvaccinated individuals showed significantly declining levels of naturally acquired SARS-CoV-2 anti-N antibodies, while anti-S levels remained stable for the time observed. While in March 2021, 25% of the study participants had at least one vaccination against SARS-CoV-2, in September of the same year, 63% were already vaccinated at least once. Study participants were assigned to the group of vaccinated as soon as they had a vaccination against SARS-CoV-2 and irrespective of the vaccine used. Therefore, some individuals of the vaccinated group might not have been vaccinated 3 months post initial blood donation or, even though already vaccinated, might not have had experienced a full immune response to the vaccination at the time the blood samples were drawn. This may explain lower values of anti-S antibodies observed within the group of vaccinated participants and similar in vitro functionality compared to unvaccinated 3 months post initial blood donation compared to later points in time.

Our data support several other studies demonstrating that there is no sex-related impact on the SARS-CoV-2 seroprevalence rate, as reviewed by Vaselli et al. [25] and Lai et al. [31]. Furthermore, our data are in line with the study from Xiang et al. who showed that the decline in SARS-CoV-2 antibody levels against the viral N-protein in hospitalized patients is independent of sex and age [32].

There are several studies indicating that the AB0 blood group might be an important factor regarding an infection with SARS-CoV-2 [33,34,35]. Concerning the infection risk, contradictory results are available: While some studies show a significantly lower infection risk for blood group 0 [36,37,38,39,40,41], others show no specific infection risk for any AB0 blood group [21,42,43]. Different cohort groups studied might explain discrepant results: While those studies documenting a lower infection risk for blood group 0 mainly focus on patients with severe disease course, studies indicating no difference focus on cases of rather mild disease course. However, there is growing evidence that blood group 0 has a lower risk for severe disease course [39,44]. Furthermore, AB individuals were demonstrated to have a higher risk for a SARS-CoV-2 infection [35,45], severe disease course including the necessity of ventilation [33,34] and increased mortality [33] compared to other AB0 blood groups.

Interestingly the data of our study revealed significantly reduced SARS-CoV-2 anti-N total antibody levels for blood group AB within the first year of infection. A similar effect was observed for anti-S antibody concentrations and in vitro functionality.

A possible explanation for the specific role of blood group AB could be that anti-A and anti-B antibodies may support virus clearance: It was demonstrated for SARS that virus particles can be glycosylated by the A-variant of AB0 glycosyltransferases, allowing anti-A antibodies to neutralize the glycosylated viral particles [46]. As speculated by Barnkob et al. [47], this could also be the case for SARS-CoV-2. Deleers et al. recently showed that recombinant SARS-CoV-2 S-protein is tagged with A- or B-antigens in vitro. The authors suggest that infectious SARS-CoV-2 particles might be tagged as well, thus supporting virus clearance [48]. These findings might explain a certain kind of protection for individuals with blood group 0 and an elevated risk for AB individuals.

It is important to note that, even though the AB0 blood group distribution in the present study reflects the normal AB0 blood group distribution among Austrian blood donors, the number of AB individuals analysed in our study is small. Further examination is required to corroborate our results.

Another interesting finding of our study is that 19% of the participants did not know about their previous SARS-CoV-2 infection. Our findings are in line with the calculation-based analytical study conducted by Johansson et al. who estimated that 24% of all infections are asymptomatic [49]. Our data further reveal no significant differences concerning the developmental course of anti-N and anti-S antibodies and in vitro antibody functionality between noticed and unnoticed infections. This is in contrast to the study conducted by Lynch et al. who demonstrated that SARS-CoV-2 antibody responses were significantly higher in patients with a severe compared to a milder disease course [50]. Another study showed that as well as severity of disease course, specific COVID-19 symptoms, namely fever, body aches and low appetite, might go along with a higher antibody response [51]. However, a direct comparison of those studies with our data is difficult as participant cohorts differed substantially with regard to symptoms and disease course. While in our study relatively healthy individuals were included, the other studies focused on critically ill COVID-19 patients, including also individuals with severe disease course up to ventilation. In addition, other factors such as sample size, detection methods applied and different points in time for measurements might lead to discrepant findings.

Providing that the infection was noticed, our study participants reported symptoms that are considered typical for a SARS-CoV-2 infection [7,52,53,54]. Even though the disease course of our participants was rather mild, some described long-lasting symptoms up to nine months post infection. Taquet et al. reported in their study that one out of three patients shows at least one symptom of long-COVID up to 6 months after the diagnosis [10]. This rate was corroborated by data of two further studies [55,56]. The risk for long-COVID appears to be higher in patients who suffered from a more severe COVID-19 illness [10]. However, our data and the studies from Gold et al. [55] and Logue et al. [56] indicate that individuals with a rather mild disease course can also be affected by long-COVID.

The present study has several strengths but also some limitations: The analysed sample size of 51,797 allows a reliable calculation of the seroprevalence rate for anti-N antibodies over time. Our study spans pre- and post-vaccination points in time and focuses on the dynamics of anti-N antibody levels, thus allowing the determination of seroprevalence rates independent of vaccination status and irrespective of currently known virus variants. Furthermore, our study participants donated blood samples at defined points in time, allowing us to follow the developmental course of antibody formation accurately. However, as children, young persons (<18 years of age), the elderly (>70 years) and adults with severely impaired health status, who are not eligible for regular blood donation, are excluded, the data cannot be extrapolated to the whole population. The developmental course of anti-N antibody levels might be different for other age groups and people with impaired health. Furthermore, it should be mentioned that people with severe long-COVID symptoms might not be healthy enough to donate blood. Thus, our data might underestimate the numbers of people suffering from prolonged symptoms after a SARS-CoV-2 infection. Even though not reported yet, we cannot completely exclude pauci-/asymptomatic reinfections in the course of our study, which might influence antibody dynamics. Although our data reveal detectability and in vitro functionality for the first year after a SARS-CoV-2 infection, it is not clear whether the longevity of antibodies is comparable to other types of coronaviruses. Thus, to date, our study is still going on with the aim to investigate SARS-CoV-2 antibody dynamics for up to 36 months.

## 5. Conclusions

In conclusion, our study shows that infection-acquired SARS-CoV-2 anti-N antibody levels are declining, but they are detectable one year after a SARS-CoV-2 infection, while anti-S levels remain stable. Antibodies detected were functional in vitro within the first year post infection. We also demonstrate that blood group AB shows significantly lower levels and in vitro functionality of SARS-CoV-2 antibodies. Our study further indicates that an asymptomatic and symptomatic COVID-19 disease course lead to a similar antibody response. Based on the investigation of more than 50,000 samples, these results provide valuable insights into the dynamics of the immune response after a SARS-CoV-2 infection in a representative cohort of adult blood donors in Central Europe.

## Figures and Tables

**Table 1:** Serological examination 3, 6 and 9 months after blood donation. Four different measurements were performed: SARS-CoV-2 anti-N total antibody screening, IgG ELISA and a quantitative ECLIA (both anti-S) and a surrogate virus neutralization test (sVNT). Data shown are median values with interquartile range for unvaccinated and vaccinated study participants. * Upper limit of the dynamic range of the applied ECLIA.

Time (Months After Donation)	SARS-CoV-2 Anti-N Total Antibody Assay ECLIA (COI)	Anti-SARS-CoV-2 IgG ELISA (Ratio Value)	Anti-SARS-CoV-2 Quantitative ECLIA (IU/mL)	Anti-SARS-CoV-2 sVNT (Inhibition in %)
Assay directed against viral				
	N-protein	S-protein	S-protein	S-protein
UNVACCINATED (*n* = 46)				
3 months	26.2 (9.5–104.0)	18.3 (15.1–20.6)	35.6 (15.2–103.0)	61.9 (49.2–79.6)
6 months	14.8 (5.3–39.1)	17.1 (16.6–18.7)	42.1 (21.7–118.0)	59.8 (45.8–79.9)
9 months	12.0 (3.7–47.0)	18.4 (17.4–21.0)	69.0 (28.4–209.0)	63.4 (42.7–81.4)
VACCINATED (*n* = 74)				
3 months	38.8 (13.0–99.3)	17.2 (15.7–19.8)	107.0 (37.2–1674.0)	79.2 (58.4–95.5)
6 months	20.7 (6.2–47.7)	17.7 (16.9–18.9)	≥2500.0 * (698.0–≥2500.0 *)	95.6 (93.8–96.2)
9 months	10.3 (3.8–39.1)	18.4 (18.3–21.0)	≥2500.0 * (1324.0–≥2500.0 *)	94.7 (94.1–95.6)

## Data Availability

Not applicable.

## References

[1] WHO (2020). WHO Director-General’s Opening Remarks at the Media Briefing on COVID-19—11 March 2020. https://www.who.int/director-general/speeches/detail/who-director-general-s-opening-remarks-at-the-media-briefing-on-COVID-19---11-march-2020.

[2] Ramesh S., Govindarajulu M., Parise R.S., Neel L., Shankar T., Patel S., Lowery P., Smith F., Dhanasekaran M., Moore T. (2021). Emerging SARS-CoV-2 variants: A review of its mutations, its implications and vaccine efficacy. Vaccines.

[3] Harvey W.T., Carabelli A.M., Jackson B., Gupta R.K., Thomson E.C., Harrison E.M., Ludden C., Reeve R., Rambaut A., Consortium C.-G.U. (2021). SARS-CoV-2 variants, spike mutations and immune escape. Nat. Rev. Microbiol..

[4] WHO (2021). Classification of Omicron (B.1.1.529): SARS-CoV-2 Variant of Concern. https://www.who.int/news/item/26-11-2021-classification-of-omicron-(b.1.1.529)-SARS-CoV-2-variant-of-concern.

[5] Wu F., Zhao S., Yu B., Chen Y.M., Wang W., Song Z.G., Hu Y., Tao Z.W., Tian J.H., Pei Y.Y. (2020). A new coronavirus associated with human respiratory disease in China. Nature.

[6] Zhu N., Zhang D., Wang W., Li X., Yang B., Song J., Zhao X., Huang B., Shi W., Lu R. (2020). A novel coronavirus from patients with pneumonia in China, 2019. N. Engl. J. Med..

[7] Machhi J., Herskovitz J., Senan A.M., Dutta D., Nath B., Oleynikov M.D., Blomberg W.R., Meigs D.D., Hasan M., Patel M. (2020). The natural history, pathobiology, and clinical manifestations of SARS-CoV-2 infections. J. Neuroimmune Pharm..

[8] Bchetnia M., Girard C., Duchaine C., Laprise C. (2020). The outbreak of the novel severe acute respiratory syndrome coronavirus 2 (SARS-CoV-2): A review of the current global status. J. Infect. Public Health.

[9] Davis H.E., Assaf G.S., McCorkell L., Wei H., Low R.J., Re’Em Y., Redfield S., Austin J.P., Akrami A. (2021). Characterizing long COVID in an international cohort: 7 months of symptoms and their impact. EClinicalMedicine.

[10] Taquet M., Dercon Q., Luciano S., Geddes J.R., Husain M., Harrison P.J. (2021). Incidence, co-occurrence, and evolution of long-COVID features: A 6-month retrospective cohort study of 273,618 survivors of COVID-19. PLOS Med..

[11] Dong Y., Dai T., Wang B., Zhang L., Zeng L.H., Huang J., Yan H., Zhang L., Zhou F. (2021). The way of SARS-CoV-2 vaccine development: Success and challenges. Signal Transduct. Target. Ther..

[12] Ling Y., Zhong J., Luo J. (2021). Safety and effectiveness of SARS-CoV-2 vaccines: A systematic review and meta-analysis. J. Med. Virol..

[13] Lipsitch M., Krammer F., Regev-Yochay G., Lustig Y., Balicer R.D. (2022). SARS-CoV-2 breakthrough infections in vaccinated individuals: Measurement, causes and impact. Nat. Rev. Immunol..

[14] Mao Y., Wang W., Ma J., Wu S., Sun F. (2021). Reinfection rates among patients previously infected by SARS-CoV-2: Systematic review and meta-analysis. Chin. Med. J..

[15] Jeffery-Smith A., Rowland T.A.J., Patel M., Whitaker H., Iyanger N., Williams S.V., Giddings R., Thompson L., Zavala M., Aiano F. (2021). Reinfection with new variants of SARS-CoV-2 after natural infection: A prospective observational cohort in 13 care homes in England. Lancet Healthy Longev..

[16] Dhillon R.A., Qamar M.A., Gilani J.A., Irfan O., Waqar U., Sajid M.I., Mahmood S.F. (2021). The mystery of COVID-19 reinfections: A global systematic review and meta-analysis. Ann. Med. Surg..

[17] Murchu E.O., Byrne P., Walsh K.A., Carty P.G., Connolly M., De Gascun C., Jordan K., Keoghan M., O’Brien K.K., O’Neill M. (2020). Immune response following infection with SARS-CoV-2 and other coronaviruses: A rapid review. Rev. Med. Virol..

[18] Siracusano G., Brombin C., Pastori C., Cugnata F., Noviello M., Tassi E., Princi D., Cantoni D., Malnati M.S., Maugeri N. (2021). Profiling antibody response patterns in COVID-19: Spike S1-reactive IgA signature in the evolution of SARS-CoV-2 infection. Front. Immunol..

[19] Petersen M.S., Hansen C.B., Kristiansen M.F., Fjallsbak J.P., Larsen S., Hansen J.L., Jarlhelt I., Perez-Alos L., Steig B.A., Christiansen D.H. (2021). SARS-CoV-2 natural antibody response persists for at least 12 months in a nationwide study from the Faroe Islands. Open Forum Infect. Dis..

[20] Wei J., Matthews P.C., Stoesser N., Maddox T., Lorenzi L., Studley R., Bell J.I., Newton J.N., Farrar J., Diamond I. (2021). Anti-spike antibody response to natural SARS-CoV-2 infection in the general population. Nat. Commun..

[21] Weidner L., Nunhofer V., Jungbauer C., Hoeggerl A.D., Gruner L., Grabmer C., Zimmermann G., Rohde E., Laner-Plamberger S. (2021). Seroprevalence of anti-SARS-CoV-2 total antibody is higher in younger Austrian blood donors. Infection.

[22] Weidner L., Gansdorfer S., Unterweger S., Weseslindtner L., Drexler C., Farcet M., Witt V., Schistal E., Schlenke P., Kreil T.R. (2020). Quantification of SARS-CoV-2 antibodies with eight commercially available immunoassays. J. Clin. Virol..

[23] Noguchi K., Gel Y.R., Brunner E., Konietschke F. (2012). nparLD: An R software package for the nonparametric analysis of longitudinal data in factorial experiments. J. Stat. Softw..

[24] R-Core-Team (2020). A Language and Environment for Statistical Computing.

[25] Vaselli N.M., Hungerford D., Shenton B., Khashkhusha A., Cunliffe N.A., French N. (2021). The seroprevalence of SARS-CoV-2 during the first wave in Europe 2020: A systematic review. PLoS ONE.

[26] Jones J.M., Stone M., Sulaeman H., Fink R.V., Dave H., Levy M.E., Di Germanio C., Green V., Notari E., Saa P. (2021). Estimated US infection- and vaccine-induced SARS-CoV-2 seroprevalence based on blood donations, July 2020–May 2021. JAMA.

[27] Raouf M., Rabeh M., Kaur S., Sharma R., Thottumkal N., Mohammed R. (2021). Seroprevalence of IgG anti-SARS-CoV-2 among voluntary blood donors in Dubai: Demographic and risk factors. Dubai Med. J..

[28] Shrotri M., Harris R.J., Rodger A., Planche T., Sanderson F., Mahungu T., McGregor A., Heath P.T., London C.G., Brown C.S. (2021). Persistence of SARS-CoV-2 N-antibody response in healthcare workers, London, UK. Emerg. Infect. Dis..

[29] Krutikov M., Palmer T., Tut G., Fuller C., Azmi B., Giddings R., Shrotri M., Kaur N., Sylla P., Lancaster T. (2022). Prevalence and duration of detectable SARS-CoV-2 nucleocapsid antibodies in staff and residents of long-term care facilities over the first year of the pandemic (VIVALDI study): Prospective cohort study in England. Lancet Healthy Longev..

[30] Chansaenroj J., Yorsaeng R., Posuwan N., Puenpa J., Wanlapakorn N., Sudhinaraset N., Sripramote M., Chalongviriyalert P., Jirajariyavej S., Kiatpanabhikul P. (2021). Long-term specific IgG response to SARS-CoV-2 nucleocapsid protein in recovered COVID-19 patients. Sci. Rep..

[31] Lai C.C., Wang J.H., Hsueh P.R. (2020). Population-based seroprevalence surveys of anti-SARS-CoV-2 antibody: An up-to-date review. Int. J. Infect. Dis..

[32] Xiang T., Liang B., Fang Y., Lu S., Li S., Wang H., Li H., Yang X., Shen S., Zhu B. (2021). Declining levels of neutralizing antibodies against SARS-CoV-2 in convalescent COVID-19 patients one year post symptom onset. Front. Immunol..

[33] Zietz M., Zucker J., Tatonetti N.P. (2020). Associations between blood type and COVID-19 infection, intubation, and death. Nat. Commun..

[34] Hoiland R.L., Fergusson N.A., Mitra A.R., Griesdale D.E.G., Devine D.V., Stukas S., Cooper J., Thiara S., Foster D., Chen L.Y.C. (2020). The association of ABO blood group with indices of disease severity and multiorgan dysfunction in COVID-19. Blood Adv..

[35] Latz C.A., Decarlo C., Boitano L., Png C.Y.M., Patell R., Conrad M.F., Eagleton M., Dua A. (2020). Blood type and outcomes in patients with COVID-19. Ann. Hematol..

[36] Leaf R.K., Al-Samkari H., Brenner S.K., Gupta S., Leaf D. (2020). ABO phenotype and death in critically ill patients with COVID-19. Br. J. Haematol..

[37] Ray J.G., Schull M.J., Vermeulen M.J., Park A.L. (2020). Association between ABO and Rh blood groups and SARS-CoV-2 infection or severe COVID-19 illness: A population-based cohort study. Ann. Intern. Med..

[38] Zhao J., Yang Y., Huang H., Li D., Gu D., Lu X., Zheng Z., Liu L., Liu T., Liu Y. (2021). Relationship between the ABO blood group and the COVID-19 susceptibility. Clin. Infect. Dis..

[39] Shokri P., Golmohammadi S., Noori M., Nejadghaderi S.A., Carson-Chahhoud K., Safiri S. (2022). The relationship between blood groups and risk of infection with SARS-CoV-2 or development of severe outcomes: A review. Rev. Med. Virol..

[40] Li J., Wang X., Chen J., Cai Y., Deng A., Yang M. (2020). Association between ABO blood groups and risk of SARS-CoV-2 pneumonia. Br. J. Haematol..

[41] Boukhari R., Breiman A., Jazat J., Ruvoen-Clouet N., Martinez S., Damais-Cepitelli A., Le Niger C., Devie-Hubert I., Penasse F., Mauriere D. (2021). ABO blood group incompatibility protects against SARS-CoV-2 transmission. Front. Microbiol..

[42] Dzik S., Eliason K., Morris E.B., Kaufman R.M., North C.M. (2020). COVID-19 and ABO blood groups. Transfusion.

[43] Boudin L., Janvier F., Bylicki O., Dutasta F. (2020). ABO blood groups are not associated with risk of acquiring the SARS-CoV-2 infection in young adults. Haematologica.

[44] Kim Y., Latz C.A., DeCarlo C.S., Lee S., Png C.Y.M., Kibrik P., Sung E., Alabi O., Dua A. (2021). Relationship between blood type and outcomes following COVID-19 infection. Semin. Vasc. Surg..

[45] Singh P.P., Srivastava A.K., Upadhyay S.K., Singh A., Upadhyay S., Kumar P., Rai V., Shrivastava P., Chaubey G., Serosurveillance Consortium BHU (2021). The association of ABO blood group with the asymptomatic COVID-19 cases in India. Transfus. Apher. Sci..

[46] Guillon P., Clement M., Sebille V., Rivain J.G., Chou C.F., Ruvoen-Clouet N., Le Pendu J. (2008). Inhibition of the interaction between the SARS-CoV spike protein and its cellular receptor by anti-histo-blood group antibodies. Glycobiology.

[47] Barnkob M.B., Pottegard A., Stovring H., Haunstrup T.M., Homburg K., Larsen R., Hansen M.B., Titlestad K., Aagaard B., Moller B.K. (2020). Reduced prevalence of SARS-CoV-2 infection in ABO blood group O. Blood Adv..

[48] Deleers M., Breiman A., Daubie V., Maggetto C., Barreau I., Besse T., Clémenceau B., Ruvoën-Clouet N., Fils J.-F., Maillart E. (2021). COVID-19 and blood groups: ABO antibody levels may also matter. Int. J. Infect. Dis..

[49] Johansson M.A., Quandelacy T.M., Kada S., Prasad P.V., Steele M., Brooks J.T., Slayton R.B., Biggerstaff M., Butler J.C. (2021). SARS-CoV-2 transmission from people without COVID-19 symptoms. JAMA Netw. Open.

[50] Lynch K.M., Cabeen R.P., Toga A.W., Clark K.A. (2020). Magnitude and timing of major white matter tract maturation from infancy through adolescence with NODDI. NeuroImage.

[51] Amjadi M.F., O’Connell S.E., Armbrust T., Mergaert A.M., Narpala S.R., Halfmann P.J., Bashar S.J., Glover C.R., Heffron A.S., Taylor A. (2021). Specific COVID-19 symptoms correlate with high antibody levels against SARS-CoV-2. Immunohorizons.

[52] Hu B., Guo H., Zhou P., Shi Z.L. (2021). Characteristics of SARS-CoV-2 and COVID-19. Nat. Rev. Microbiol..

[53] Mehta O.P., Bhandari P., Raut A., Kacimi S.E.O., Huy N.T. (2020). Coronavirus disease (COVID-19): Comprehensive review of clinical presentation. Front. Public Health.

[54] Tsai P.-H., Lai W.-Y., Lin Y.-Y., Luo Y.-H., Chen H.-K., Chen Y.-M., Lai Y.-C., Kuo L.-C., Chen S.-D., Chang K.-J. (2021). Clinical manifestation and disease progression in COVID-19 infection. J. Chin. Med. Assoc..

[55] Gold J., Okyay R., Licht W., Hurley D. (2021). Investigation of long COVID prevalence and its relationship to Epstein-Barr virus reactivation. Pathogens.

[56] Logue J.K., Franko N.M., McCulloch D.J., McDonald D., Magedson A., Wolf C.R., Chu H.Y. (2021). Sequelae in adults at 6 months after COVID-19 infection. JAMA Netw. Open.

